# Eclampsia among adolescent mothers in low- and middle-Income countries

**DOI:** 10.1371/journal.pgph.0003890

**Published:** 2025-03-26

**Authors:** Laura van der Krogt, Alexandra Ridout, Nicola Vousden, Hannah L. Nathan, Paul T. Seed, Betty Sam Midwife, Mariama Momoh Midwife, Tom Sesay, Francis Smart, Mrutunjaya Bellad, Bellington Vwalika, Andrew Shennan

**Affiliations:** 1 Division of Women’s Health, King’s College London, Women’s Health Academic Centre, St Thomas’ Hospital, London, United Kingdom; 2 National Perinatal Epidemiology Unit, Nuffield Department of Population Health, University of Oxford, Oxford, United Kingdom; 3 Welbodi Partnership, Freetown, Sierra Leone; 4 Ministry of Health, Freetown, Sierra Leone; 5 Jawaharlal Nehru Medical College, Belgaum, Karnataka, India; 6 University of Zambia, Lusaka, Zambia; Harvard Medical School, UNITED STATES OF AMERICA

## Abstract

Everyday, approximately 800 pregnant women die from preventable causes, with 95% of these deaths occurring in low- and middle-income countries. Adolescent mothers are disproportionally affected. Hypertensive disorders, including eclampsia, contribute to around 20% of direct maternal deaths, many of which are preventable with simple, cost-effective interventions. This study aims to quantify the risk of eclampsia among adolescent mothers in low- and middle-income countries. This secondary analysis used data from three large multi-center studies within the CRADLE programme of work, conducted across ten regions in Sub-Saharan Africa, India, and Haiti. Data on eclampsia and maternal age were prospectively collected from routine sources and active case finding. The incidence rates of eclampsia were calculated, and the relative risk of eclampsia in adolescents were calculated and compared to non-adolescent mothers. Among 601,343 deliveries, 3,098 cases of eclampsia were recorded (0·51%). The incidence of eclampsia varied widely, from 22 per 10,000 deliveries in Zambia to 142 per 10,000 deliveries in Sierra Leone, a 6·5-fold variation. Adolescents accounted for 34% of eclampsia cases (1065/3098). The relative risk of eclampsia in adolescents compared to older mothers ranged from 1·50 (95% CI 1.32 to 1.83) to 3·45 (95% CI 2.71 to 4.41) across sites (p<0.0001). Adolescent mothers in low- and middle- income countries studied faced a significantly higher risk of eclampsia compared to older mothers. The reproducibility of this finding across diverse settings and time periods highlights the vulnerability of adolescent mothers to health inequalities associated with hypertensive disorders. Further research is needed to explore mechanisms underlying eclampsia in this group, independent of the severity of maternal or fetal disease. Developing targeted interventions and strategies to improve maternal and perinatal outcomes in this high-risk group should be a priority.

## Introduction

In 2020, one woman died every two minutes from preventable causes related to pregnancy. The vast majority of these deaths, over 95%, occurred in low- and middle-income countries [1]. Hypertensive disorders of pregnancy, including eclampsia and pre-eclampsia, are among the leading preventable causes of maternal death, accounting for an estimated 21% of maternal mortality in these settings [2].

Pre-eclampsia is significantly associated with adverse maternal and perinatal outcomes. These include stroke, preterm birth, placental abruption, fetal growth restriction, and stillbirth, all of which have long-term implications for both mothers and their offspring [3–8]. Women who experience severe pre-eclampsia at a young age have a higher risk of recurrence in future pregnancies and are more likely to develop chronic hypertension and heart failure in the future [9].

The effects of pre-eclampsia extend beyond the maternal population. Children born from pregnancies complicated by pre-eclampsia have a three-fold increased risk of developing hypertension by the age of 20 [10], highlighting the intergenerational impact of this condition.

Eclampsia is defined as the occurrence of generalized convulsions with increased blood pressure, in the absence of epilepsy or another condition predisposing to convulsions [11]. Although the exact etiology remains unclear, it is related to pre-eclampsia and represents the severe end of the disease spectrum. Prolonged severe hypertension can results in loss of autoregulation of the cerebral vasculature, resulting in cerebral hyperperfusion, oedema, and disruption of the blood brain barrier [12]. It is associated with significant risk of maternal mortality.

The incidence of eclampsia in high-income settings has declined dramatically over the past half century, largely due to improved antenatal care, early initiation of preventative measures, and effective treatment, including timely delivery of the baby [13]. However, outcomes for women and girls in low-income settings remain in stark contrast. The incidence of eclampsia in the United Kingdom is 2.7 per 10,000 deliveries, compared to 142 per 10,000 deliveries in Sierra Leone [2,11], underscoring the stark disparities in maternal healthcare between these contexts.

Globally, approximately 11% of all pregnancies occur in adolescent mothers, defined as those younger than 20 years old, according to the World Health Organisation [14]. Each year, an estimated 21 million girls aged 15 to 19 years in developing regions become pregnant [14]. Adolescents are well-documented to have higher risk of maternal and perinatal complications compared with older women, including an increased incidence of pre-eclampsia [3–8]. Sociodemographic factors, such as limited support, poor access to healthcare and low attendance at antenatal care, are throught to contribute to this increased risk [14–17].

Emerging evidence suggests that the clinical presentation of pre-eclampsia and eclampsia may differ in adolescents. Severe disease in this group is often observed at lower blood pressure thresholds compared to older mothers [7,8]. The underlying mechanism driving these differences remains unclear, though they may involve distinct pathophysiological processes. Factors such as the maternal immune system and uterine immaturity are hypothesized to contribute to adverse placentation and the increased susceptability of eclampsia in adolescent mothers [18–21].

We aim to determine whether the incidence and risk of eclampsia among adolescent mothers aged 13 to 19 in low- and middle-income countries differ from those observed in non-adolescent mothers.

## Methods

### Ethics statement

CRADLE-2 was approved by the Stellenbosch University Ethics Committee (N14/06068), University of Cape Town Ethics Committees (410/2014) and the University of the Free State Ethics Committee (230408-011). Informed written consent from participants and institutional level consent was obtained. CRADLE-3 was granted by the King’s College London (LRS-14/15-1484) and in all countries before the start of the trial (appendix). Institutional-level consent on behalf of the cluster was obtained. CRADLE-5 was approved from King’s College London (HR/DP-21/22-27014) and the Sierra Leone Ethics and Research Council. Informed, written consent was obtained from participants.

This is a secondary analysis of data from the CRADLE Programme, which was conducted in South Africa, Ethiopia, Malawi, Sierra Leone, Uganda, Zambia, Zimbabwe, India, and Haiti. The CRADLE programme aimed to develop (CRADLE-2), implement (CRADLE-3) and evaluate the scale-up (CRADLE-5) of a novel vital signs alert device and focused obstetric emergency training package, with the aim of reducing maternal morbidity and mortality associated with hypertensive disorders of pregnancy, obstetric haemorrhage, and sepsis [22–24].

CRADLE-2 was a prospective observational cohort study of women with pre-eclampsia admitted to three tertiary facilities in South African between January 2015 and May 2016 [22]. All women with a diagnosis of pre-eclampsia were eligible for inclusion, with no exclusion criteria [22].

CRADLE-3 was a stepped-wedge, cluster-randomised controlled trial introducing the CRADLE intervention across ten sites in eight countries – Ethiopia, Sierra Leone, Zimbabwe, Uganda, Zambia, Malawi, India, and Haiti - between April 2016 and November 2017 [23].

CRADLE-5 was a stepped-wedge, cluster-randomised controlled trial evaluating the scale-up of the CRADLE device in eight districts of Sierra Leone from January 2022 to June 2023 [24]. In CRADLE-3 and CRADLE-5 all pregnant women or those within 6-weeks postpartum presenting for maternity care were included, with no exclusion criteria [23,24].

The total number of deliveries and cases of eclampsia were prospectively collected at each site during the CRADLE-2, CRADLE-3 and CRADLE-5 trials. United Nations World Fertility Data was used to identify the adolescent pregnancy rate at each site during each specified time period, which was then used to estimate the total number of adolescent pregnancies [25].

The incidence of eclampsia (per 10,000 deliveries) and the proportion of eclampsia cases among adolescents was then calculated to interrogate the relationship between age at delivery and incidence of eclampsia (Table 1). CRADLE-2 data was not included in this analysis as this cohort pertained only to women already diagnosed with pre-eclampsia. The data from all three studies was used to calculate the relative risk (RR) of eclampsia in adolescent mothers compared to non-adolescents (Table 3). Statistical significance was defined as p <0.05, and results presented with 95% confidence intervals (CI).

**Table 1:** Total number of deliveries and the adolescent pregnancy rate for each study site.

	Total Number of Deliveries	Number of Deliveries in adolescent mothers (<20 years old)	Adolescent Pregnancy Rate as Percentages
South Africa (2015–2016)	1547	.	.
Ethiopia (2016–2017)	35429	4397	12.41
Haiti (2016–2017)	12910	1730	11.60
Sierra Leone (2016–2017)	23806	4423	18.58
India (2016–2017)	22876	1144	5.00
Zimbabwe (2016–2017)	38383	6897	17.97
Zambia (2016–2017)	150345	29092	19.35
Uganda (2016–2017)	188319	34481	18.31
Malawi (2016–2017)	62165	9325	20.79
Sierra Leone (2022–2023)	63563	13348	21.38

The data available for this sub-analysis was specific to age; confounders such as body mass index, parity and maternal medical conditions were not controlled for. Data was accessed on January 22, 2023, and no participant-identifiable information was accessed during or after data collection. Statistical analysis was conducted with Stata version 17 (StataCorp, College Station, TX).

## Results

There were 601,343 deliveries across the CRADLE programme. The adolescent pregnancy rate varied up to 4·2-fold between countries, ranging from 5% in India compared to 21% in Sierra Leone. A summary of the number of deliveries and adolescent pregnancy rates across all sites is provided in Table 1.

**Fig 1:**
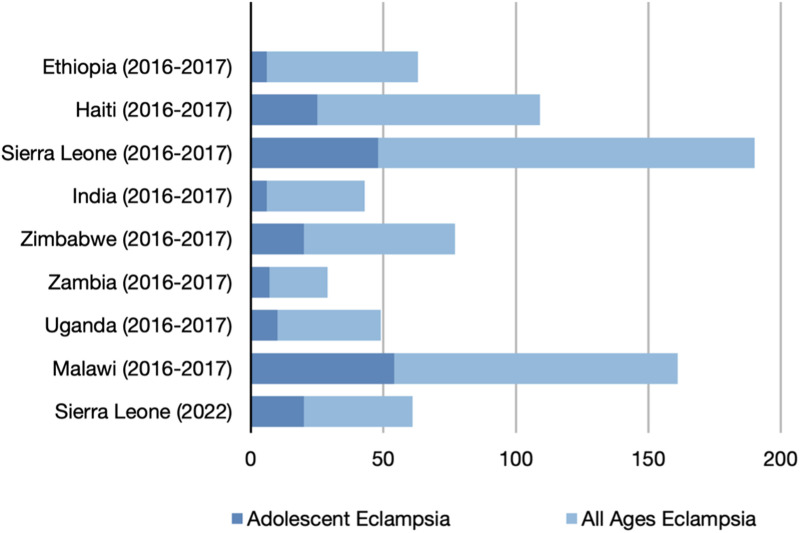
Comparison of incidence of eclampsia in adolescents and all ages per 10,000 deliveries.

There were 3,098 cases of eclampsia, representing an overall incidence of 0·51%. Adolescent mothers accounted for one-third of all eclamptic fits (34%, 1072/3098). In the whole cohort, the incidence of eclampsia varied up to 6·5-fold between sites, from 22 per 10,000 deliveries in Zambia to 142 per 10,000 deliveries in Sierra Leone. Among adolescent mothers, the incidence of eclampsia varied approximately 9-fold, from 6 per 10,000 deliveries in Ethiopia to 54 per 10,000 deliveries in Malawi. Table 2 summarises the cases of eclampsia across all study sites. Fig 1 illustrates the incidence of eclampsia and adolescent eclampsia across the CRADLE-3 and CRADLE-5 trial sites.

**Table 2:** Eclampsia cases across each study site.

	Total number of women experiencing eclampsia	Eclampsia in adolescent mothers (<20 years)	Incidence of eclampsia (per 10,000 deliveries)	Incidence of Adolescent Eclampsia (per 10,000 deliveries)
South Africa (2015–2016)	147	54	.	.
Ethiopia (2016–2017)	203	20	57	6
Haiti (2016–2017)	125	37	84	25
Sierra Leone (2016–2017)	338	115	142	48
India (2016–2017)	85	13	37	6
Zimbabwe (2016–2017)	218	78	57	20
Zambia (2016–2017)	331	106	22	7
Uganda (2016–2017)	726	188	39	10
Malawi (2016–2017)	666	337	107	54
Sierra Leone (2022–2023)	259	124	41	20

**Fig 2:**
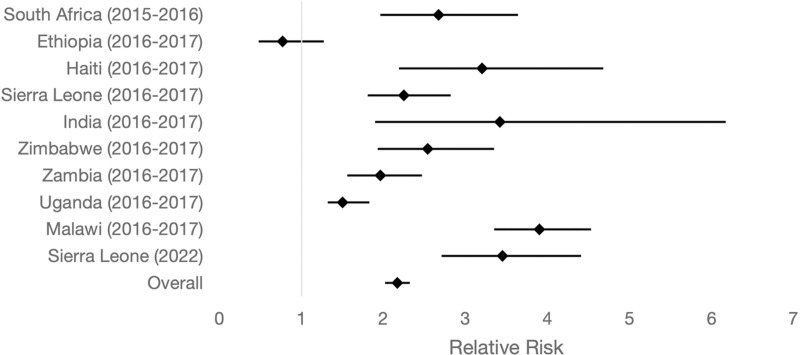
A forest plot showing the relative risk of eclampsia in adolescents compared to non-adolescents mothers.

The RR of eclampsia in adolescent mothers was significantly higher than in non-adolescents across most sites. The RR ranged from 1·50 in Uganda to 3·90 in Malawi. Ethiopia was the only exception, with an RR of 0.77, indicating a lower risk of eclampsia in adolescents compared to non-adolescents (Table 3 and Fig 2). In India, the RR of eclampsia in adolescents was 3.42, but the CI was wide (1.90 to 6.17). This likely reflects the relatively small number of women experiencing eclampsia in this cohort (35 total cases, of which 13 occurred in adolescents) (Tables 2 and Table 3).

**Table 3:** Relative Risk of eclampsia in adolescents compared to eclampsia in non-adolescents across all study sites.

	Relative Risk	95% Confidence Interval	Significance (P-value)
South Africa (2015–2016)	2.67	1.96 to 3.64	<0.0001
Ethiopia (2016–2017)	0.77	0.48 to 1.27	0.26
Haiti (2016–2017)	3.20	2.19 to 4.68	<0.0001
Sierra Leone (2016–2017)	2.25	1.81 to 2.82	<0.0001
India (2016–2017)	3.42	1.90 to 6.17	<0.0001
Zimbabwe (2016–2017)	2.54	1.93 to 3.35	<0.0001
Zambia (2016–2017)	1.96	1.56 to 2.47	<0.0001
Uganda (2016–2017)	1.50	1.32 to 1.83	<0.0001
Malawi (2016–2017)	3.90	3.35 to 4.53	<0.0001
Sierra Leone (2022–2023)	3.45	2.71 to 4.41	<0.0001

## Discussion

### Principal findings

This study highlights a significantly increased incidence of eclampsia among adolescent mothers across low- and middle-income countries, with consistent findings across multiple sites and time periods.

### Results in the context of what is known

Adolescent pregnancies remain common worldwide, particularly in low-resource settings, and are associated with increased maternal and perinatal morbidity and mortality [3,4,14]. While prior research has established increased rates of pre-eclampsia in adolescents [6–9], this study provides robust evidence of a heightened risk of eclampsia, the most severe manifestation of hypertensive disordered of pregnancy. It is not known why some women progress to eclampsia, and others do not [9]. Eclampsia’s pathogenesis remains poorly understood, with mechanisms of progression from pre-eclampsia likely influenced by biological, immunological and sociocultural factors [3–8,15,18–21].

Biological hypotheses include poor trophoblastic invasion and defective remodeling of myometrial spiral arteries [25], leading to abnormal placentation. The immune maladaptation hypothesis suggests that the response of the maternal immune system to novel and recently exposed paternally derived fetal antigens drives abnormal placentation [18,21]. This is particularly relevant in primiparous adolescents with limited exposure to paternal antigens due to short paternity durations or infrequent sexual activity [18].

Uterine immaturity may also contribute to defective deep placentation [19,20]. It has been proposed that regular cycles of menstrual shedding and tissue regeneration are essential for the uterus to develop plasticity and stress resistance to allow for tissue re-modeling and vascular adaptation from the deeply invading trophoblast. This concept is known as ‘menstrual preconditioning’ and is supported by the observation that ‘gynecological age’ or years at conception minus age at menarche, is a stronger predictor of adverse pregnancy outcome than chronological age in adolescents [19,20].

Sociodemographic factors, such as stigma, insufficient social support, delayed care-seeking, and poor antenatal care engagement, exacerbate risks for adolescents in low-resource settings [14,15,17]. This is especially relevant in low- and middle-income countries where these issues are compounded by poverty, lack of education and scarce employment opportunities [17].

It has been suggested that in addition to accessible, inclusive and adolescent-friendly health care provision, community engagement and education initiatives are methods which can address sociodemographic issues [17]. Interventions addressing these barriers, such as mentoring schemes and community engagement programmes, have shown promise in improving maternal and neonatal outcomes, as demonstrated in Sierra Leone [15]. Potential mechanisms of action include relationship building, advocacy, community building as well as educational, social, and economic empowerment, which could be integrated into healthcare [16].

### Clinical implications

This work builds upon existing knowledge that adolescent pregnancies are associated with increased adverse maternal outcomes [3,4], reinforcing the need for heightened surveillance for hypertensive disordered in pregnant adolescents. Current guidelines do not account for adolescents’ unique risk profiles, including their tendency to develop severe disease at lower blood pressure thresholds [7,8]. Given our findings and those of Olaya-Garay et al., adolescent-specific monitoring strategies, such as recognizing smaller baseline blood pressure increases as significant, may be warranted and should be recognized within national and international practice guidelines, particularly in low-resource settings where adolescent pregnancies are prevalent [3–8,14]. Care for the adolescent should be holistic and guidelines may need to be adapted.

Interventions to diagnose and manage pre-eclampsia early, before complications such as eclampsia occur, are essential. These interventions are not only critical but also likely to be cost-effective, particularly in health care systems with limited resources. Evidence from the CRADLE-4 trial supports this, demonstrating that planned early delivery of women with late preterm pre-eclampsia in a low- or middle-income countries led to a reduction in stillbirths and severe maternal hypertension [26]. This highlights the cost-effectiveness of early detection and intervention strategies, and emphasises the potential for scalable solutions in resource-limited settings [27].

### Research implications

Further research is needed to establish appropriate definitions of hypertension in this cohort, including evaluating blood pressure increases relative to baseline as a diagnostic threshold. Studies must also investigate the pathogenesis of pre-eclampsia and eclampsia in adolescents and whether this differs from the adult population. If differences are found, novel biomarkers or therapeutic targets should be prioritized given the significant associated risks. Further exploration of sociocultural and systemic barriers to care is essential for tailoring interventions to this vulnerable population.

### Strengths and limitations of this study

This study utilised a large, prospectively collected dataset, ensuring robust clinical endpoints and consistency across diverse geographic and temporal contexts (3–5,14). (3–5,14). To our knowledge, it represents one of the largest reported data sets of eclampsia in adolescents.

A limitation of our data set was that we could only examine the incidence of eclampsia. Future research should investigate the mechanisms underpinning this, including the prospective collection of blood pressure measurements using a validated blood pressure device such as the CRADLE Vital Signs Alert device. Additionally, the timing of eclampsia should be investigated, with strategies from improved antenatal or postnatal monitoring and escalation where needed. It would be warranted to explore the interactions of other risk factors for pre-eclampsia and eclampsia, such as body mass index, parity and maternal medical conditions, in both adolescents and non-adolescents.

Another limitation is the potential underreporting of adolescent pregnancies, which may underestimate the true burden of eclampsia in this population. Anecdotal evidence suggests that stigma and fear of retribution may lead adolescents to misreport their age or avoid formal healthcare, compounding challenges in data accuracy.

Despite these limitations, the findings highlight critical disparities in maternal health outcomes for adolescents and underscore the need for targeted interventions and research.

Whilst the overall incidence of eclampsia in Sierra Leone has decreased significantly, from 1.4% in 2016-2017 (CRADLE-3 data) to 0.4% in 2022 (CRADLE-5 data), aligning with reductions in the maternal mortality ratio (which fell from 1,682 per 100,000 in 2000 to 443 per 100,000 in 2020, as reported by the World Health Organisation [27], these improvements are not reflected in adolescent eclampsia rates. Strikingly, teenagers still account for one-third of all cases [28].

Underreporting may explain why the higher relative risk of eclampsia in adolescents compared to non-adolescents across all countries studied, except Ethiopia (RR 0.77 (95% CI 0.48-1.27), p = 0.26). The overall incidence of eclampsia (57 per 10,000, or 0.5%) was consistent with other studies, which report rates of 0.3 to 0.71% [28,29]. However, adolescents accounted for 9% of all eclamptic cases, lower than the 17% reported elsewhere [29], and significantly lower than the 30% observed across all other CRADLE study sites. Possible unknown protective lifestyle or dietary factors may contribute to this discrepancy, which warrants further investigation [28].

## Conclusion

Adolescent mothers face a significantly higher risk of eclampsia across studied low and lower middle-income countries. This finding is reproducible across multiple sites and time periods, emphasizing the urgent need to address inequalities in maternal healthcare for this vulnerable population with hypertensive disorders. Further research needs to explore potential mechanisms driving this increased risk of eclampsia, independent of the severity of maternal or fetal disease, and to develop targeted strategies for prevention, early detection and management.

## References

[1] Trends in maternal mortality 2000 to 2020: estimates by WHO, UNICEF, UNFPA, World Bank Group and UNDESA/Population Division [Internet]. 2023 [cited 2023 Oct 27]. Available from: https://www.who.int/publications/i/item/9789240068759

[2] Vousden N, Holmes E, Seed PT, Gidiri MF, Goudar S, Sandall J, et al. Incidence and characteristics of pregnancy-related death across ten low- and middle-income geographical regions: secondary analysis of a cluster randomised controlled trial. BJOG. 2020;127(9):1082–9. doi: 10.1111/1471-0528.16309 32383337

[3] Ganchimeg T, Ota E, Morisaki N, Laopaiboon M, Lumbiganon P, Zhang J, et al. Pregnancy and childbirth outcomes among adolescent mothers: a World Health Organization multicountry study. BJOG. 2014;121 Suppl 1:40–8. doi: 10.1111/1471-0528.12630 24641534

[4] Kawakita T, Wilson K, Grantz K, Landy H, Huang C, Gomez-Lobo V. Adverse maternal and neonatal outcomes in adolescent pregnancy. Journal of Pediatric and Adolescent Gynecology. 2016;29(2):130–6. 26327561 10.1016/j.jpag.2015.08.006 PMC4886236

[5] Bakwa-Kanyinga F, Valério EG, Bosa VL, Alfama CO, Sperb M, Capp E, et al. Adolescent pregnancy: Maternal and fetal outcomes in patients with and without preeclampsia. Pregnancy Hypertens. 2017;10:96–100. doi: 10.1016/j.preghy.2017.06.009 29153698

[6] Macedo TCC, Montagna E, Trevisan CM, Zaia V, de Oliveira R, Barbosa CP, et al. Prevalence of preeclampsia and eclampsia in adolescent pregnancy: A systematic review and meta-analysis of 291,247 adolescents worldwide since 1969. Eur J Obstet Gynecol Reprod Biol. 2020;248:177–86. doi: 10.1016/j.ejogrb.2020.03.043 32283429

[7] Vigil-De Gracia P, Olaya-Garay S, Mata Hernández C, Cabrera S, Reyes-Tejada O, Asturizaga-Soto P, et al. Blood pressure changes in adolescents with preeclampsia: A multicentre, case-control study in Latin American hospitals. Journal of Obstetrics and Gynaecology Canada. 2021;43(1):50–7. 33041217 10.1016/j.jogc.2020.06.024

[8] Olaya-Garay SX, Velásquez-Trujillo PA, Vigil-De Gracia P. Blood pressure in adolescent patients with pre-eclampsia and eclampsia. International Journal of Gynecology and Obstetrics. 2017 Sep 1;138(3):335–9. 28602034 10.1002/ijgo.12237

[9] Sibai BM, el-Nazer A, Gonzalez-Ruiz A. Severe preeclampsia-eclampsia in young primigravid women: subsequent pregnancy outcome and remote prognosis. Am J Obstet Gynecol. 1986;155(5):1011–6. doi: 10.1016/0002-9378(86)90336-4 3777042

[10] Davis EF, Lewandowski AJ, Aye C, Williamson W, Boardman H, Huang RC, et al. Clinical cardiovascular risk during young adulthood in offspring of hypertensive pregnancies: insights from a 20-year prospective follow-up birth cohort. [cited 2023 Oct 17]; Available from: http://bmjopen.bmj.com/10.1136/bmjopen-2015-008136 PMC4480003 26105032

[11] Knight M, . Eclampsia in the United Kingdom 2005. BJOG. 2007;114(9):1072–8. doi: 10.1111/j.1471-0528.2007.01423.x 17617191

[12] Bergman L, Torres-Vergara P, Penny J, Wikström J, Nelander M, Leon J, et al. Investigating maternal brain alterations in preeclampsia: the need for a multidisciplinary effort. Curr Hypertens Rep. 2019;21(9):72. doi: 10.1007/s11906-019-0977-0 31375930

[13] Berhan Y, Berhan A. Should magnesium sulfate be administered to women with mild pre-eclampsia? A systematic review of published reports on eclampsia. J Obstet Gynaecol Res. 2015;41(6):831–42. doi: 10.1111/jog.12697 25833188

[14] World Health Organisation. Adolescent Pregnancy [Internet]. 2023 [cited 2023 Oct 17]. Available from: https://www.who.int/news-room/fact-sheets/detail/adolescent-pregnancy

[15] Turienzo CF, November L, Kamara M, Kamara P, Goodhart V, Ridout A, et al. Innovations to reduce maternal mortality and improve health and wellbeing of adolescent girls and their babies in Sierra Leone. Lancet Child Adolesc Health. 2023;7(3):151–3. doi: 10.1016/S2352-4642(22)00322-4 36442481

[16] STAGE Group. Transforming women’s, children’s, and adolescents’ health and wellbeing through primary health care. Lancet. 2023;402(10413):1606–8. doi: 10.1016/S0140-6736(23)01909-8 37722398

[17] Sabet F, Prost A, Rahmanian S, Al Qudah H, Cardoso MN, Carlin JB, et al. The forgotten girls: the state of evidence for health interventions for pregnant adolescents and their newborns in low-income and middle-income countries. Lancet. 2023;402(10412):1580–96. doi: 10.1016/S0140-6736(23)01682-3 37837988

[18] Dekker G, Robillard P-Y. Pre-eclampsia: Is the immune maladaptation hypothesis still standing? An epidemiological update. J Reprod Immunol. 2007;76(1–2):8–16. doi: 10.1016/j.jri.2007.03.015 17493684

[19] Brosens I, Muter J, Gargett CE, Puttemans P, Benagiano G, Brosens JJ. The impact of uterine immaturity on obstetrical syndromes during adolescence. Am J Obstet Gynecol. 2017 Nov 1;217(5):546–55. 28578177 10.1016/j.ajog.2017.05.059

[20] Brosens I, Muter J, Ewington L, Puttemans P, Petraglia F, Brosens JJ, et al. Adolescent preeclampsia: pathological drivers and clinical prevention. Reprod Sci. 2019;26(2):159–71. doi: 10.1177/1933719118804412 30317927

[21] Levron Y, Dviri M, Segol I, Yerushalmi G, Hourvitz A, Orvieto R, et al. The “immunologic theory” of preeclampsia revisited: A lesson from donor oocyte gestations. Am J Obstet Gynecol. 2014;211(4):383.e1-383.e5. 10.1016/j.ajog.2014.03.044 24657130

[22] Nathan H, Seed P, Hezelgrave N, De Greeff A, Lawley E, Conti-Ramsden F, et al. Maternal and perinatal adverse outcomes in women with pre-eclampsia cared for at facility-level in South Africa: a prospective cohort study. J Glob Health. 2018;8(2):e2018001. 10.7189/jogh.08-020401 PMC6076583 30140431

[23] Vousden N, Lawley E, Nathan H, Seed P, Gidiri M, Goudar S, et al. Effect of a novel vital sign device on maternal mortality and morbidity in low-resource settings: a pragmatic, stepped-wedge, cluster-randomised controlled trial. Lancet Glob Health. 2019;7(3):e347-56. doi: 10.1016/S2214-109X(19)30045-0 30784635 PMC6379820

[24] Ridout AE, Moses FL, Herm-Singh S, Turienzo CF, Seed PT, Goodhart V, et al. CRADLE-5: a stepped-wedge type 2 hybrid implementation-effectiveness cluster randomised controlled trial to evaluate the real-world scale-up of the CRADLE Vital Signs Alert intervention into routine maternity care in Sierra Leone-study protocol. Trials. 2023;24(1):590. doi: 10.1186/s13063-023-07587-4 37723530 PMC10506317

[25] Chaiworapongsa T, Chaemsaithong P, Yeo L, Romero R. Pre-eclampsia part 1: current understanding of its pathophysiology. Nat Rev Nephrol. 2014;10(8):466–80. doi: 10.1038/nrneph.2014.102 25003615 PMC5893150

[26] Beardmore-Gray A, Vousden N, Silverio SA, Charantimath U, Katageri G, Bellad M, et al. Planned early delivery for late preterm pre-eclampsia in a low- and middle-income setting: a feasibility study. Reproductive Health. 2021;18(1). 10.1186/s12978-021-01159-y PMC8173959 34078408

[27] WHO Sierra Leone annual report for 2022 | WHO | Regional Office for Africa [Internet]. 2022 [cited 2023 Dec 1]. Available from: https://www.afro.who.int/countries/sierra-leone/publication/whosierra-leone-annual-report-2022

[28] Wagnew M, Meazaw M, Chojenta C, Taddele T, Loxton D. Preeclampsia and eclampsia: Its burden and distribution across facilities in Ethiopia. Pregnancy Hypertension. 2022;29(1):64–71. 35797744 10.1016/j.preghy.2022.06.006

[29] Getaneh Y, Fekadu E, Jemere AT, Mengistu Z, Tarekegn GE, Oumer M. Incidence and determinants of adverse outcomes among women who were managed for eclampsia in the University of Gondar Comprehensive Specialized Hospital, Northwest Ethiopia. BMC Pregnancy Childbirth. 2021;21(1):734. doi: 10.1186/s12884-021-04199-1 34715798 PMC8555341

